# Distinct Tryptophan Catabolism and Th17/Treg Balance in HIV Progressors and Elite Controllers

**DOI:** 10.1371/journal.pone.0078146

**Published:** 2013-10-16

**Authors:** Mohammad-Ali Jenabian, Mital Patel, Ido Kema, Cynthia Kanagaratham, Danuta Radzioch, Paméla Thébault, Réjean Lapointe, Cécile Tremblay, Norbert Gilmore, Petronela Ancuta, Jean-Pierre Routy

**Affiliations:** 1 Chronic Viral Illness Service, McGill University Health Centre, Montreal, Quebec, Canada; 2 Research Institute, McGill University Health Centre, Montreal, Quebec, Canada; 3 Department of Laboratory Medicine, University Medical Center Groningen, University of Groningen, Groningen, The Netherlands; 4 CHUM Research Centre (CRCHUM), Montreal, Quebec, Canada; 5 Department of Medicine, Université de Montreal, Montreal, Quebec, Canada; 6 Department of Microbiology, Infectiology and Immunology, Faculty of Medicine, Université de Montreal, Montreal, Quebec, Canada; 7 Division of Hematology, McGill University Health Centre, Montreal, Quebec, Canada; New York University, United States of America

## Abstract

Tryptophan (Trp) catabolism into immunosuppressive kynurenine (Kyn) by indoleamine 2,3-dioxygenase (IDO) was previously linked to Th17/Treg differentiation and immune activation. Here we examined Trp catabolism and its impact on Th17/Treg balance in uninfected healthy subjects (HS) and a large cohort of HIV-infected patients with different clinical outcomes: ART-naïve, Successfully Treated (ST), and elite controllers (EC). In ART-naïve patients, increased IDO activity/expression, together with elevated levels of TNF-α and sCD40L, were associated with Treg expansion and an altered Th17/Treg balance. These alterations were normalized under ART. In contrast, Trp 2,3-dioxegenase (TDO) expression was dramatically lower in EC when compared to all other groups. Interestingly, EC displayed a distinctive Trp metabolism characterized by low Trp plasma levels similar to ART-naïve patients without accumulating immunosuppressive Kyn levels which was accompanied by a preserved Th17/Treg balance. These results suggest a distinctive Trp catabolism and Th17/Treg balance in HIV progressors and EC. Thus, IDO-induced immune-metabolism may be considered as a new inflammation-related marker for HIV-1 disease progression.

## Introduction

Chronic HIV-1 infection is characterized by progressive depletion of total CD4+ T-cells and persistent immune activation, events that are only partially controlled by antiretroviral therapy (ART). Immune activation is associated with increased production of inflammatory soluble factors, further contributing to immune dysfunction [1]. Immune stimulators including interferon (IFN)γ [2], cytotoxic T-lymphocyte antigen-4 (CTLA-4) ligation [3] and Toll-like receptor (TLR) stimulation [4] induce intracellular indoleamine 2,3-dioxygenase (IDO) by macrophages and dendritic cells (DCs) [5,6]. IDO catabolizes the essential amino acid Tryptophan (Trp) into an immunosuppressive metabolite, Kynurenine (Kyn), that limits immune responses in cancers and chronic viral infections and/or induces immune tolerance during pregnancy[5-11]. Another enzyme that catabolizes Trp is Tryptophan 2,3-dioxygenase (TDO) which is mainly expressed in the liver as well as other tissues including the brain, uterus and skin [12-15].

Among T-cell subsets, regulatory T-cells (Tregs), play a pivotal role in peripheral tolerance and pathogenesis of cancer and chronic viral infections [16]. Indeed, Tregs were shown to suppress effector T-cells activation and function [17]. Forkhead box P3 (FoxP3), the master regulator of Treg function, can influence the balance between Treg and T-helper 17 (Th17) cells. Th17 cells play a critical role in maintaining the integrity of mucosal immunity against pathogens [18-21]. HIV-1 infection is characterized by a rapid Th17 cell depletion associated with an expansion of Tregs owing to cellular immune activation and/or low CD4+ T-cell counts [18,19]. The impaired Th17/Treg balance in HIV-1 infection has a deleterious effect on gut mucosal immunity and fuels immune activation by enhancing microbial translocation [9,22,23]. It has been recently shown that IDO-induced Trp catabolism promotes T-cell differentiation into Treg *versus* Th17 cells through FoxP3 over-expression [9,24,25]. Importantly, for both Simian immunodeficiency virus (SIV) and HIV-1 infections, the altered Th17/Treg balance in blood and mucosal tissues is directly linked to a sustained increase of IDO activity via IFN-γ signaling and TLR ligation [2,18]. Findings by Favre et al. in HIV-infected subjects indicate that elevated IDO activity is associated with enhanced microbial translocation and faster disease progression [2,18]. 

Herein, we assessed IDO-induced Trp catabolism in relation with Th17/Treg balance in the largest cohort of HIV-infected patients ever studied in this context, including a remarkable subset of patients called elite controllers (EC) who achieve long-term control of viremia and disease progression in the absence of ART [26]. Our results provide evidence that IDO-induced Trp catabolism into Kyn induces a harmful effect on the Th17/Treg ratio that may subsequently contribute to enhanced microbial translocation during HIV-1 infection. Importantly, EC compared to ART-Successfully Treated (ST) and healthy subjects (HS) displayed a distinctive Trp catabolism characterized by similar Kyn/Trp ratios despite significantly lower plasma Trp levels, dramatically reduced TDO expression, and preserved IDO expression and Th17/Treg ratios. Thus, new therapeutic interventions modulating the IDO-mediated Trp catabolism may help limit disease progression in HIV-infected subjects. 

## Materials and Methods

### Study population

Peripheral blood mononuclear cells (PBMC) and plasma were collected from untreated ART-naïve patients (n=96), ART-successfully treated (ST, n=88) and healthy subjects (HS, n=50) at the Chronic Viral Illness Service, McGill University Health Centre (MUHC), Montreal, QC, Canada. Samples from elite controllers (EC) (n=19) were obtained from the FRQ-S slow progressor cohort, Montreal, QC, Canada (Table 1). To avoid the influence of other factors that could modulate Trp levels, all subjects were accounted for seasonal variation and nutritional status (body mass index, albumin and cholesterol levels) [27,28]. Plasma levels of Trp, Kyn and 3-Hydroxykynurenine (3OH-Kyn) were measured in all 253 subjects. Levels of inflammatory markers, sCD14, Treg and Th17 cell frequency, IDO-1, IDO-2 and TDO mRNA expression were evaluated in a random subset of n=14 per study group. 

**Table 1:** Clinical and virological characteristics of study groups.

**Characteristics**	**Study population N=253**			
	**ST (n=88)**	**ART- naïve (n=96)**	**EC (n=19)**	**HS (n=50)**
**Age (years) [Mean ± SD (range)]**	48.4±9.4 (29-81)	39.2±9.2 (23-58)	49.0±7.1 (40-62)	45.0±9.9 (19-64)
**Male [n (%)]**	73 (83%)	76 (79%)	9 (47%)	35 (70%)
**Risk factors**:				
Men Who Have Sex with Men [n (%)]	59 (67%)	58 (60%)	6 (32%)	NA
Heterosexual [n (%)]	15 (17%)	22 (23%)	10 (53%)	NA
Endemic [n (%)]	11 (13%)	12 (13%)	2 (11%)	NA
Injection drug users [n (%)]	3 (3%)	4 (4%)	1 (5%)	NA
**Time since HIV-1 diagnosis** (**years**) [Mean ± SD (range)]	10.1±5.5 (2-24)	4.2±5.2 (0-23)	13.1±4.1 (4-20)	NA
**CD4 T-cell count (cells/µL**) [Mean ± SD range)]	531.3±267.4 (37-1282)	416.1±189.2 (3-1110)	617.8±209.4 (417-1341)	812.6±273.0(281-1559)
**CD8 T-cell count (cells/µL**)[Mean ± SD (range)]	785.8±332.6 (153-2013)	761.9±365.2 (237-1933)	559.7±307.0 (162-1198)	390.9±188.5 (95-843)
**CD4:CD8 ratio** [Mean ±SD (range)]	0.75±0.42 (0.08-2.06)	0.64±0.38 (0.01-12.09)	1.35±0.61 (0.35-2.72)	2.45±0.96 (0.38-4.34)
**VL** (**log10copies/mL**) [Mean ±SD (range)]	< 1.6	4.0±0.87 (1.73-6.35)	< 1.6	NA
**Time of undetectable VL (years**)[Mean ± SD (range)]	3.64±2.7 (1-10)	NA	12.8±7.0 (7-20)	NA
**Time since start of ART** (**years**) [Mean ± SD (range)]	8.1±4.7 (1-20)	NA	NA	NA

These include ART-successfully treated (ST), ART-naïve, Elite controllers (EC) and Healthy subjects (HS). Results are shown as mean ± standard deviation (SD) and (Range), NA: not applicable, VL: Viral Load.

### Ethics statement

This study, using PBMC and plasma samples from HIV-infected and uninfected subjects, was conducted in compliance with the principles included in the Declaration of Helsinki. This study received approval from the Ethical Review Board of the McGill University Health Center, Montreal, Canada. All blood donors provided written informed consent of their participation to the study.

### Measurement of the plasma concentration of Tryptophan and its catabolites

Plasma levels of Trp, Kyn and 3OH-Kyn were measured by an automated on-line solid-phase extraction-liquid chromatographic-tandem mass spectrometric (XLC-MS/MS) method as previously reported [29]. Briefly 250μL plasma was mixed with 50 μL deuterated internal standard working solution (300 μmol/L in diluted acetic acid for Trp and 5 μmol/L for Kyn and 3OH-Kyn) and diluted with 200 μL water. The samples were placed into the autosampler, which picks up 50μL of the sample leading it to the solid-phase extraction chromatography (SPE) cartridge. The sample was washed on the SPE cartridge where the washed cartridge extract was eluted into the high-performance liquid chromatography (HPLC) column. The binary gradient system consisted of mobile phase A (0.2% formic acid in water) and mobile phase B (acetonitrile). During this step, chromatographic procedure separated Trp, Kyn and 3OH-Kyn. The HPLC column effluent was then led into a mass spectrometer operated in positive ionization MRM mode to protonate the ions, and quantitatively detect selected masses. Finally, to quantify the amino acid metabolites, the area of specific mass peaks was measured and related to concentration of calibration curves of the respective metabolites.

### *In vitro* characterization of Th17 cells

PBMCs were thawed in a 37°C water bath and cultured in 48-well culture plates at 0.5-1 × 10^6^ cells/mL per well. These assays were performed with no stimulation as a negative control or stimulation with PMA (50 ng/mL) and ionomycin (1 μg/ml) for 2 hours at 37°C and 5% CO_2_. BFA (2 μg/ml) was then added to block cytokine secretion and cells were kept in culture for 18 hours at 37°C and 5% CO_2_.. The following day, the cells were transferred into FACS tubes, centrifuged at 1500rpm for 5 minutes and stained for detection of IL-17A. IFNγ staining was used as a positive control for the assay since it is highly expressed by T-lymphocytes. PBMCs underwent surface staining for 20 minutes at 4°C. Cells were fixed using Cytofix/CytoPerm for 20 minutes at 4°C following permeabilization using Perm/Wash 1X subsequentl to intracellular staining with IL-17A and IFNγ at 4°C for 30 minutes. Excess antibody was removed by washing with Perm/Wash 1X following a final wash with FACS buffer before flow cytometry acquisition.

### Flow cytometry

Detection of surface and intracellular markers was performed by a four-laser LSRII flow cytometer (BD Bioscience, Mississauga, ON, CA). The following Abs were used: CD3-Pacific blue, CD4-PercpCy5.5, CD4-PECy5, CD8-Alexa700, CD25-PE, CD127-PECy7, CD38-APC, and IFN-γ-Alexa700 (BD Bioscience, Mississauga, Ontario, CA); CD45RA-ECD was from Beckman Coulter (Mississauga, Ontario, CA) and IL-17-PE, CD8-APCeFluor780 and FOXP3Alexa488 were from eBioscience (San Diego, USA). Magnetic beads coated with anti-rat and anti-mouse Abs were used as positive/negative controls for compensation calculation (BD Bioscience, Mississauga, Ontario, CA). The viability marker Vivid AmCyan (Invitrogen, Burlington, Ontario, CA) was used to exclude dead cells from analysis. Data was exported and analyzed using FlowJo software v7.6.5. Tregs were characterized as CD3^+^CD4^+^CD25^high^CD127^low^FoxP3^high^ and Th17 cells as CD3^+^CD4^+^IL-17a^+^ upon PMA/ionomycin stimulation.

### Quantification of IDO-1, IDO-2 and TDO mRNA

Total RNA from PBMCs were extracted with RNeasymini kits (Qiagen, Hilden, Germany). cDNA was synthesized from total RNA (500 ng) with oligo-dT and random hexamer primers (Applied Biosystems, Carlsbad, CA, USA), using an Omniscript reverse transcriptase kit (Qiagen). Then, specific mRNAs were amplified using the following primers: IDO-1 (forward: CGCTGTTGGAAATAGCTTCTTGC; revers: CTTCCCAGAACCCTTCATACACC), IDO-2 (forward: CGTCATAGCAAGGAAAGTGGTGA; revers: CCCTCAGGGAAGGTGCTGAG), TDO (forward: TCAGTTGCTGACTTCTCTTATGG; revers: CAGTTGATCGCAGGTAGTGATAG), 18s ribosomal RNA (forward: ATCAACTTTCGATGGTAGTCG; revers: TCCTTGGATGTGGTAGCCG). Quantitative real-time RT-PCR was performed by Rotorgene 3000 thermal cycler (Corbett Life Science, Sydney, Australia) and Quantitect Sybr Green PCR kit (Qiagen). The cycling conditions were 10 min at 95°C, then 35 cycles of 30s at 94°C, 45s at 60°C, and 30s at 72°C for 18s rRNA. For IDO-1, IDO-2, and TDO amplifications, 40 cycles were performed with 60s annealing at 55°C, 52°C, and 56°C, respectively, followed by 30s at 72°C. A ﬁnal melting curve was obtained from 72 to 95°C. The expression of each gene was quantified relative to the housekeeping gene 18s rRNA.

### Measurement of plasma levels of sCD14 and IL-6

Plasma levels of sCD14, as well as IL-6 were measured using commercially available human sCD14 and IL-6 ELISA kits (both from R&D systems Minneapolis, MN, USA), according to the manufacturer’s instructions. 

### Multiplex quantification of inflammatory plasmatic cytokines

Prior to analysis, all plasma samples were treated 10:1 with 5% TritonX-100 for one hour in order to inactivate the HIV-1 virus. Plasma levels of selected soluble factors implicated in IDO induction, including IFNγ, TNF-α, IL-1β and sCD40L were measured in duplicates using a MILLIPLEX MAP magnetic bead kit according to the manufacturer’s instructions (Millipore, Billerica, MA, USA). A broad range of standards were run in duplicates along with quality controls provided by the manufacturer to ensure the proper functioning of the kit. Mean fluorescence intensities for each analyte in each sample were detected using the MAGPIX instrument (Luminex) and the results were analyzed using the Millipore Analyst software version 3.5.5 to obtain the protein concentration of each soluble factor.

### Statistical analysis

Statistical analyses were performed using GraphPad Prism software version 5. One-way ANOVA or Kruskal-Wallis tests were performed for comparisons between study groups according to the sample size. Unpaired t-tests or Mann-Whitney *U* tests were used for comparison of two non-paired study variables according to the sample size. The Pearson rank correlation test was used to identify association amongst study variables. All results are presented in Mean±SD throughout the manuscript. 

## Results

### Plasma concentrations of Trp and its catabolites in HIV-infected patients with different clinical outcomes

It has been shown by Favre et al. that increased IDO activity affects T-cell function and is linked to HIV-1 disease progression [2]. To confirm and expand these findings, plasma levels of Trp and its key catabolites, Kyn and 3OH-Kyn, were measured in 253 study subjects. 

**Figure 1:**
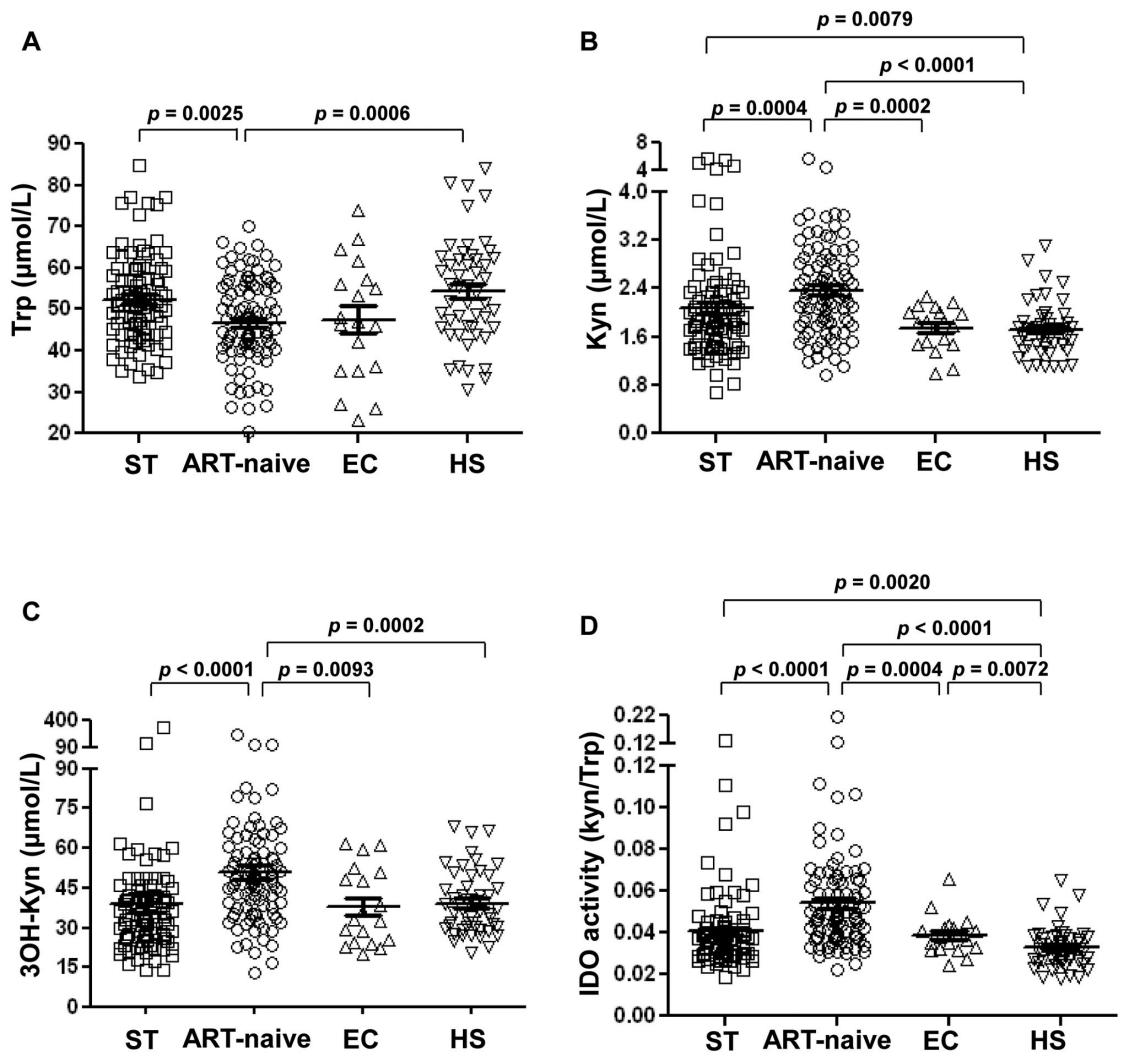
Tryptophan catabolism is elevated in HIV-infected subjects with disease progression but not elite controllers. Plasma levels of (**A**) tryptophan (Trp), (**B**) kynurenine (Kyn), (**C**) and 3 -hydroxykynurenine were measured by XLC-MS/MS in ART-successfully treated (ST, n=88), ART-naïve (n=96), elite controllers (EC, n=19), and healthy subjects (HS, n=50), (**D**) IDO activity in shown, defined as the Kyn/Trp ratio. Statistical analysis was provided by ANOVA tests followed by unpaired t-tests used for comparison of two variables.

ART-naïve compared to ST and HS demonstrated significantly lower Trp levels (ART- naïve 46.60±11.24 vs. ST: 52.39±11.62 and HS: 54.46±12.68 μmol/L; Figure 1A). In contrast, EC who spontaneously control viral load (VL) in absence of ART had comparable Trp levels to ART-naïve subjects (47.40±14.45 μmol/L). Levels of the Trp catabolite Kyn were significantly elevated in ART-naïve subjects compared to all other groups (ART-naïve: 2.37±0.76 *vs.* ST: 2.08±0.91, EC: 1.74±0.36, HS: 1.72±0.44 μmol/L; Figure 1B). Similar results were obtained for the Kyn byproduct 3OH-Kyn (ART-naïve: 50.96±26.09, ST: 39.26±33.49, EC: 37.84±14.37 and HS: 39.24±11.84 nmol/L; Figure 1C). Correspondingly, IDO enzyme activity (Kyn/Trp ratio) was also elevated in ART-naïve subjects compared to all other groups (ART-naïve: 0.054±0.026 *vs.* ST: 0.041±0.018, EC: 0.039±0.011, HS: 0.033±0.010 μmol/L; Figure 1D). By contrast, EC maintained comparable levels of Trp catabolites and IDO activity when compared to ST and HS possibly suggesting a particular Trp catabolism in these patients, which may be related to their spontaneous ability to control viral replication. No significant difference was observed in Trp, Kyn and 3OH-Kyn levels between EC with or without protective HLA alleles, HLA B57 and B27[30] (*p*>0.05 for all comparisons, data not shown). 

**Figure 2:**
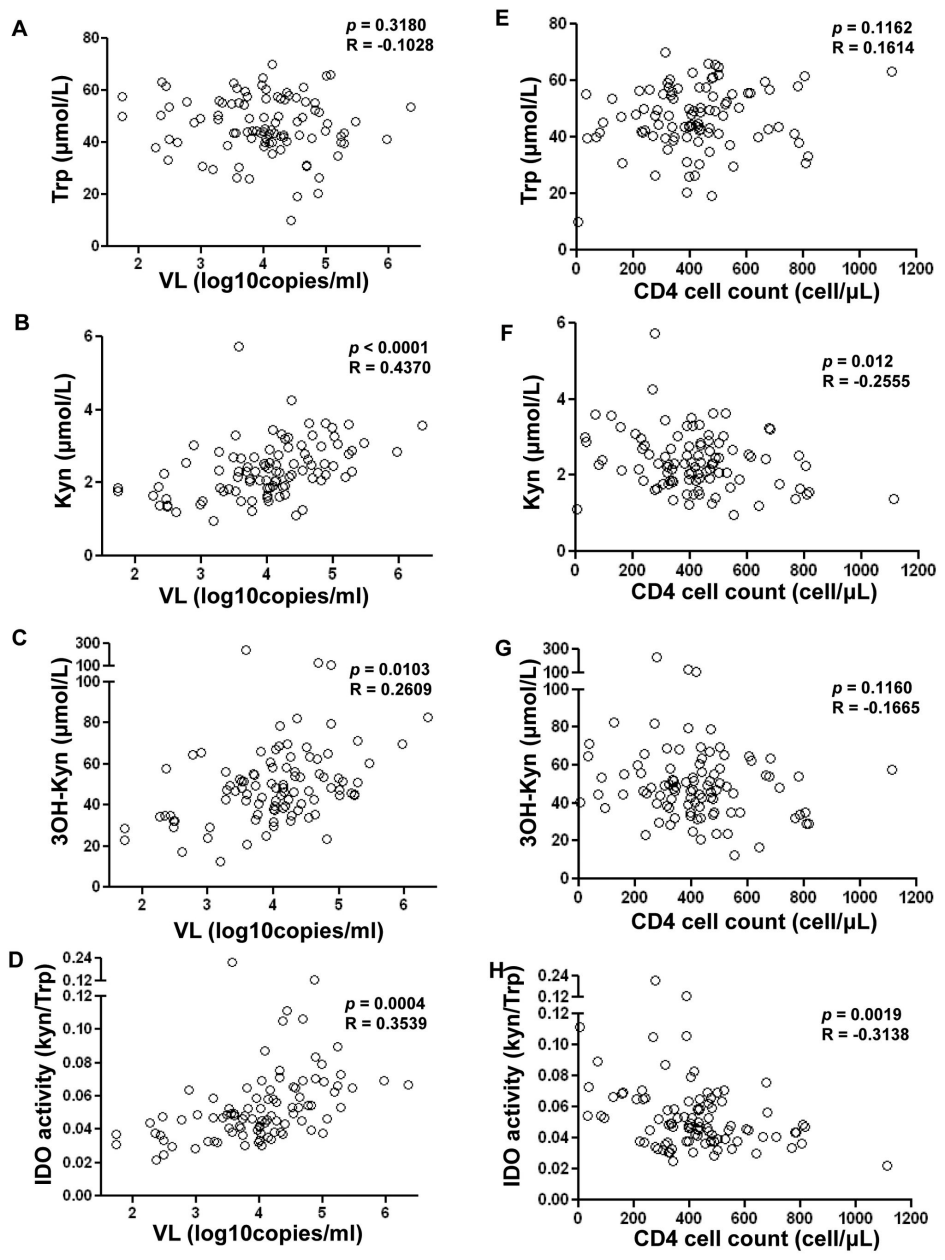
Tryptophan catabolites are associated with higher HIV-1 viral load (VL) and lower CD4+ T-cell counts in ART-naïve patients. Association between plasmatic HIV-1 viral load and plasmatic (**A**) tryptophan (Trp) (**B**) kynurenine (Kyn) (**C**) 3-hydroxykynurenine (3OH-Kyn) and (**D**) IDO activity as defined by Kyn/Trp and CD4+ T-cell counts and plasmatic (**E**) tryptophan (**F**) kynurenine (**G**) 3-hydroxykynurenine and (**H**) IDO activity (Kyn/Trp ratio) in ART-naïve patients (n=96). Pearson rank correlation was used for statistical analysis.

In ART-naïve patients, while Trp levels were not associated with VL, levels of Kyn, 3OH-Kyn and IDO enzymatic activity (Kyn/Trp ratio) were positively correlated with plasma VL (Figure 2A-D). Absence of correlation was observed between Trp and CD4+ T cell counts whereas Kyn, as well as the Kyn/Trp ratio, were negatively correlated with CD4+ T-cell counts (Figure 2E-G).

### IDO enzymatic activity and its impact on Th17/Treg imbalance in HIV-1 infection

**Figure 3:**
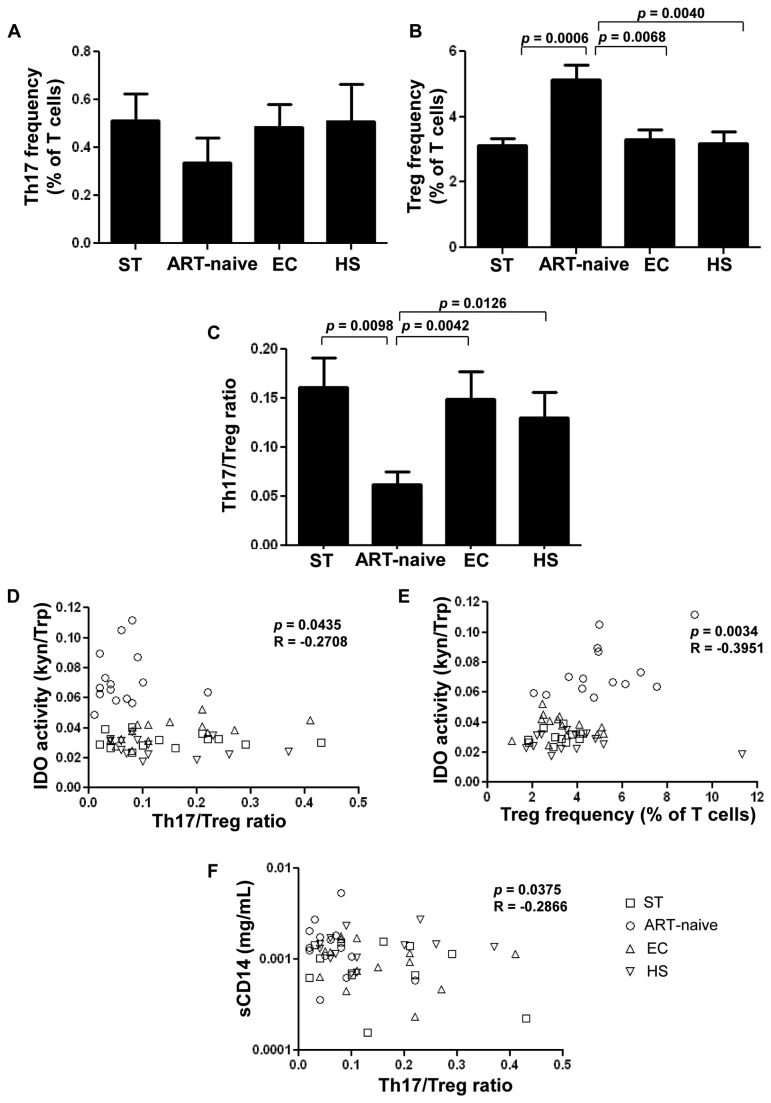
Th17/Treg imbalance in HIV-1 infection correlates with IDO enzyme activity. (**A**) Th17 cells (CD4^+^IL-17^+^) frequency in study groups following PMA/Ionomycine *in* *vitro* Stimulation (**B**) Frequency of Treg (CD4^+^CD25^high^CD127^low^FoxP3^high^ cells) in study groups *ex* *vivo* . (**C**) Th17/Treg ratio in study groups. (**C**) Th17/Treg ratio in study groups. (**D**) Correlation between IDO enzymatic activity defined by Trp/Kyn ratio and Th17/Treg ratio in study groups. (**E**) Association between IDO enzymatic activity and Treg frequency in study groups. (**F**) Correlation between bacterial translocation marker sCD14 and Th17/Treg ratio in study groups. ST: ART-successfully treated, ART-naïve, EC: elite controllers, and HS: healthy subjects. n=14 per study group. Statistical analysis was provided by Kruskal-Wallis test followed by Mann-Whitney *U* test for comparison of two variables.

A decreased Th17/Treg ratio has been previously associated with elevated plasma Trp catabolites in HIV-infected patients [2]. We therefore examined the expression of these T-cell subsets in the peripheral blood in each study group. Our results showed a trend towards decreased Th17 frequency in ART-naïve subjects compared to all other groups, (ART-naïve: 0.337±0.406% *vs.* ST: 0.512±0.424%; EC: 0.486±0.346% and HS: 0.510±0.580%). However, statistical significance was not reached between groups (Figure 3A). In contrast, the Treg frequency was significantly increased in ART-naïve patients compared to all other groups (ART-naïve; 5.13±1.82% *vs.* ST; 3.12±0.84% EC; 3.31±1.17% and HS; 3.20±1.22%; Figure 3B). Consequently, ART-naïve subjects exhibited the lowest Th17/Treg ratios compared to all other groups (ART-naïve: 0.062±0.052 *vs.* ST: 0.161±0.115 EC: 0.150±0.104 and HS: 1.3±0.10; Figure 3C). Notably, Th17/Treg ratios in EC and ST were comparable to those in HS (Figure 3C). 

Moreover, IDO is a critical molecule, which stimulates Tregs while blocking the reprogramming of Tregs into Th17 [9,25]. Our results showed IDO enzymatic activity (Kyn/Trp ratio) was associated with Th17/Treg ratio (Figure 3D) as well as with Treg frequency (Figure 3E). As sCD14 has been shown to be a reliable marker of microbial translocation [23], we showed that the imbalance of Th17/Treg was inversely correlated to elevated plasmatic sCD14 (Figure 3F).

### mRNA expression of the Trp catabolizing enzymes IDO-1, IDO-2 and TDO in HIV-infected patients

**Figure 4:**
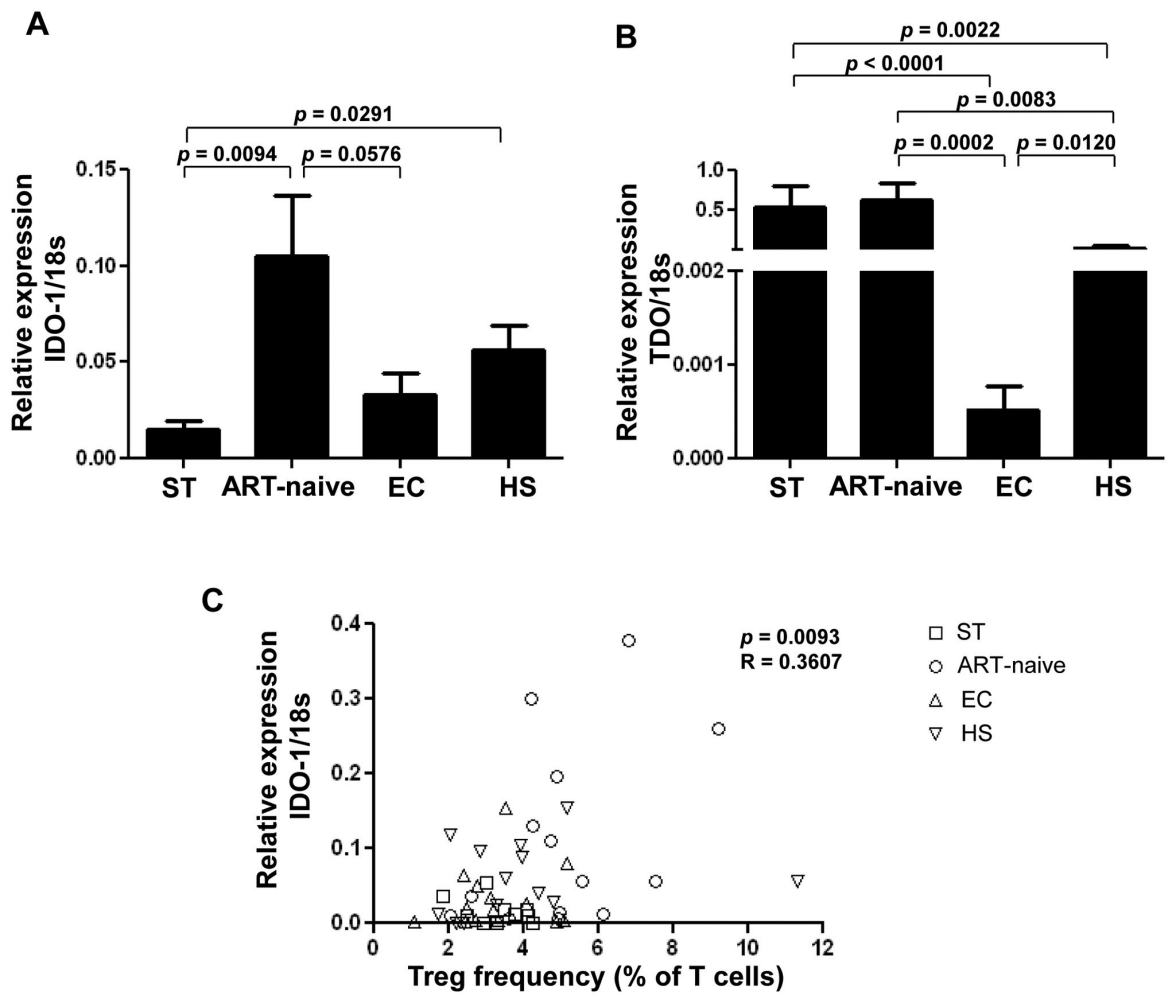
Trp catabolizing enzyme IDO-1 mRNA expression is associated with Treg frequency in HIV-1 infected patients with different clinical outcomes. (**A**) Real-time quantitative RT-PCR was used to quantify IDO-1 mRNA and (**B**) TDO expression relative to the 18s ribosomal RNA from PBMCs of study groups. (**C**) Association between IDO enzymatic activity and Treg frequency in study groups. ST: ART-successfully treated, ART-naïve, EC: elite controllers, and HS: healthy subjects. N=14 per study group. Statistical analysis was provided by Kruskal-Wallis test followed by Mann-Whitney *U* test for comparison of two variables. Pearson rank test was used for the correlation.

To further investigate the molecular basis for increased IDO enzymatic activity, we quantified IDO-1 and IDO-2 mRNA relative expression in PBMCs. ART-naïve patients demonstrated significantly higher levels of IDO-1 mRNA expression, compared to ST, EC and HS (0.113±0.124 *vs.* 0.015±0.018, 0.033±0.043 and 0.056±0.049). Interestingly, EC exhibited IDO-1 mRNA levels comparable to HS and ST (Figure 4A). In addition to IDO-1, we also evaluated levels of IDO-2 mRNA, another enzyme responsible for Trp catabolism into Kyn but expressed in different cell subsets suggesting these enzymes are not functionally redundant [31]. Unlike IDO-1 expression, IDO-2 mRNA relative expression was unchanged between study groups (ST: 0.061±0.155, ART-naïve: 0.098±0.281, EC: 0.065±0.245 and HS: 0.065±0.238, *p*>0.05 for all comparisons; data not shown). 

Another enzyme, TDO, regulates systemic Trp [12]. Our results demonstrated that levels of TDO were dramatically lower in EC when compared to all other groups (EC: 0.0005±0.001 *vs.* ST: 0.529±0.986, ART-naïve: 0.618±0.834 and HS: 0.030±0.082). Of note, TDO levels were also significantly lower in HS when compared to ART-naïve subjects and to ST (Figure 4B). Our results also showed a trend between TDO mRNA expression and Kyn/Trp ratios when evaluated in all study subjects (*p*=0.06, R=0.261, data not shown). Such a correlation was not observed when evaluated in each study group separately (*p*>0.05 for all comparisons, data not shown).

We then studied the correlation between IDO-1 and TDO mRNA levels with plasma HIV-RNA VL and CD4+ T-cell counts in ART-naïve subjects. We found a trend for a positive correlation between IDO-1 mRNA and plasma VL (*p*=0.0761, R=0.4714; data not shown). In contrast, IDO-1 mRNA expression was negatively correlated with CD4+ T-cell count in ART-naïve subjects (*p*=0.0051, R=-0.6821; data not shown). No correlation was fond between TDO expression and VL and CD4+ T-cell count (not shown). We also observed a positive association between Treg frequency and IDO-1 mRNA expression (Figure 4C). 

### IDO enzymatic activity is associated with elevated levels of TNF-α and sCD40L in HIV-infected patients

In cancer patients, inflammatory changes indicate that IDO may be induced by soluble factors including IFNγ, TNF-α, IL-1β, soluble CD40 ligand (sCD40L) and CTLA-4 ligation to CD80/CD86 expressed by DC [5,32,33]. *In vitro* stimulation of DCs by IFNγ promotes IDO-1 while IL-1β, IL-6, and TNF-α enhance IFNγ ability to promote IDO-activity [2]. 

**Figure 5:**
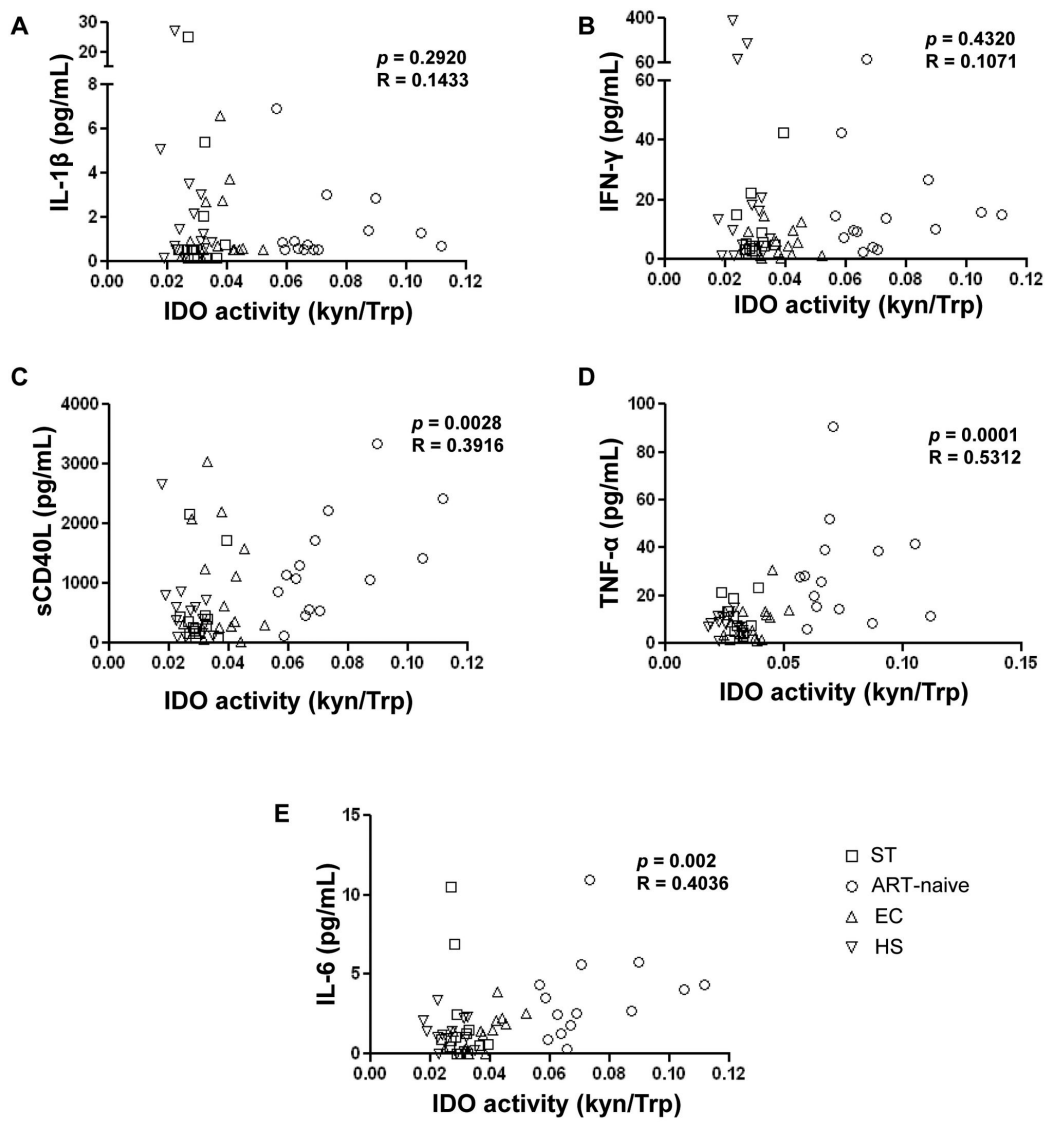
Correlations between IDO enzymatic activity and levels of inflammatory soluble factors implicated in IDO induction. Associations between IDO activity (Kyn/Trp ratio) and plasmatic levels of (**A**) IL-1β (**B**) IFNγ (**C**) sCD40L (**D**) TNF-α and (**E**) IL-6 in ST: ART-successfully treated, ART-naïve, EC: elite controllers, and HS: healthy subjects. n=14 per study group. Pearson rank correlation was used for statistical analysis.

sCD40L plasma levels were significantly higher in ART-naïve subjects compared to ST (Table 2). In contrast, EC had similar sCD40L levels compared to ST. Levels of IFN-γ and TNF-α were significantly elevated in ART-naïve subjects compared to EC (Table 2). Moreover, IL-6 was significantly higher in ART-naïve patients compared to all other groups. Interestingly, among the soluble factors implicated in IDO induction, sCD40L and TNF-α as well as IL-6, but not IL-1β and IFNγ, were positively correlated to elevated IDO enzymatic activity when evaluated in all study subjects (Figure 5). Such a correlation between soluble factors and IDO activity was only observed for sCD40L in ART-naïve patients (*p*=0.0270, R=0.5880) as well as HS (*p*=0.0435, R=-0.5457) and for IL-6 in EC (*p*=0.0090, R=0.6684) when evaluated in each study group separately (Table S1). A similar positive correlation was also observed between plasma sCD40L, TNF-α and IL-6 levels and levels of IDO-1 mRNA expression when evaluated in all study subjects (*p*=0.0015, R=0.4289; *p*=0.03, R=0.2969 and *p*<0.0001, R=0.5939 respectively; Table S2). Such a correlation between soluble factors and IDO-1 mRNA expression was only observed for sCD40L and IL-6 in the ART-naïve group when evaluated in each study group separately (*p*=0.0175, R=0.6223 and *p*=0.0181, R=0.6196 respectively; Table S2).

**Table 2:** Levels of inflammatory soluble factors in HIV-infected patients with different clinical outcomes.

	**ST**	**ART-naive**	**EC**	**HS**	***p* value**
**IL-1β (pg/ml)**	2.644±1.777	1.546±0.4678	1.516±0.4909	3.410±1.876	0.1225
**IFN-γ (pg/ml)**	9.239±2.979	18.670±5.822	5.172±1.269	30.86±16.37	0.0224
**sCD40L (pg/ml)**	502.7±167.2	1305.0±234.0	968.7±252.4	601.3±173.8	0.0127
**TNF-α (pg/ml)**	22.01±11.53	29.94±5.93	9.18±2.08	7.67±1.08	0.0008
**IL-6 (pg/ml)**	0.5807±0.2033	8.0930±5.0010	1.3360±0.7890	9.7060±5.9110	0.0034

Plasma levels of inflammatory soluble factors IL-1β, IFN-γ, sCD40L, TNF-α and IL-6 were measured by Multiplex are shown in Mean±SD pg/ml. Kruskall-Wallis test was used for statistical analysis. ST: ART-successfully treated, ART-naive, EC: elite controllers, and HS: healthy subjects. n=14 per study group.

## Discussion

Numerous studies provided evidence for an association between HIV disease progression and increased plasma levels of inflammatory factors such as IDO-induced Trp catabolism [1,2,34]. IDO catabolizes Trp into Kyn and is involved in control of inflammatory signals providing protection for tissue damage and inducing immune tolerance [5,6]. The Trp catabolite Kyn has immunosuppressive properties suppressing T-cell functions and proliferation [7,35]. In this study, we evaluated Trp levels and its catabolites in a well-defined cohort of HIV-infected patients with different clinical outcomes. Trp levels were significantly decreased in HIV-infected adults whose infection was uncontrolled (ART-naïve subjects) when compared to those whose infection was successfully controlled pharmacologically or who were uninfected confirming prior reports [10,11]. ART-naïve also demonstrated the highest levels of Kyn, its byproduct (3OH-Kyn), and IDO enzymatic activity (Trp/Kyn ratio). In contrast, levels of Trp catabolites declined in ART-treated subjects as did IDO enzymatic activity. In addition, Trp catabolites and Kyn/Trp ratio were associated with both elevated plasma viral load and low CD4+ T-cell counts. Thus, ART normalized Trp catabolism in HIV-infected subjects by reducing IDO enzymatic activity. It is of interest that increased Trp catabolism in HIV infection is implicated in neuropathology [36] and disease progression [34]. 

EC constitute a minority of HIV-infected patients that are able to control viral replication and preserve CD4+ T-cell counts over time in the absence of ART [26]. EC have efficient immune responses and activation linked in part to HLA genetic determinism [26,37]. In SIV infection, SIV controllers display much lower IDO expression in blood, lymph nodes and gut associated lymphoid tissues as compared to pathogenic SIV-infection [38,39]. Interestingly, our results demonstrate that Trp levels were similarly low in EC and ART-naïve subjects whereas levels of Trp catabolites and Kyn/Trp ratio were significantly lower and were similar to those found in ST or HS. IDO expression can be triggered by Forkhead box O3 (FOXO3) via induction of superoxide dismutase and blockade of peroxynitrite formation [40]. Thus, silencing FOXO3a expression inhibits Trp catabolism mediated by CTLA-4-Ig [41]. Furthermore, down-regulation of FOXO3a transcriptional activity in EC represents a molecular signature which can help protect survival of memory T-cells and slow disease progression [42,43]. Our findings suggest a link between distinctive Trp catabolism in EC and a consistently lower FOXO3a expression and IDO activity in these patients. Our results showed no difference in Trp, Kyn and 3OH-Kyn levels between EC with or without protective HLA alleles B57 and B27, indicating that mechanisms other than those governed by the adaptive immunity are regulating Trp catabolism in EC. Altogether, we have observed a distinctive pattern of Trp catabolism in EC; whereby they do not seem to accumulate immunosuppressive Trp catabolites and do not progresses to immune dysfunction or tolerance. While unexplained, these changes suggest a new avenue for HIV control. 

The Th17/Treg balance plays a dominant role in the maintenance of mucosal immunity [9,16]. These cells are derived from common progenitor cells and their differentiation pathways are reciprocally modulated upon immune activation during HIV-1 infection, resulting in bacterial translocation [9,18,20,21]. We observed a lower Th17/Treg ratio in ART-naïve subjects when compared to the other study groups. This was associated with a lower Th17 frequency and an enrichment of Treg frequency, together with higher levels of microbial translocation marker sCD14. One potential explanation is that Trp catabolites induce Tregs and reduce the generation of Th17 cells [2,9,24,25]. Our results also show such a relationship where IDO enzymatic activity (Kyn/Trp ratio) was associated with a decrease in Th17/Treg ratio, driven mainly by an increase in Treg frequency. This suggests that the immunomodulatory enzyme IDO, through its Trp deprivation and/or an increase in its immunosuppressive catabolites, may be an important pathway that can maintain chronic inflammation in HIV-1 infection.

Moreover, we found that IDO-1 mRNA expression was significantly increased in ART-naïve subjects and was associated with elevated viral load, low CD4+ T-cell count and importantly elevated Treg frequency. We also evaluated IDO-2 enzyme mRNA expression which is also expressed in antigen-presenting cells (not as widely expressed as IDO-1) and catabolizes the same enzymatic step as IDO-1 [31]. We observed slightly lower levels of IDO-1 mRNA expression in total PBMCs from ST and EC compared to HS possibly due to a redistribution of DCs, the main producers of IDO-1, into lymphoid tissues in HIV-infected patients. Thus, quantification of IDO-1 mRNA in the FACS-sorted leucocyte populations may help to better evaluate the nature of these changes. Unlike the changes in IDO-1 expression, IDO-2 mRNA expression was not different between study groups, thus highlighting the pivotal immunosuppressive role of IDO-1 in HIV-1 infection. TDO, expressed mostly by the liver and also by the brain, uterus, and skin, is responsible for regulating systemic Trp [12-15]. TDO expression is constitutively increased in Alzheimer's disease [44] and various human tumors and is equally capable of suppressing antitumor immune responses [13,45]. Our results showed that ART-naïve and ST patients have significantly higher TDO mRNA expression when compared to HS. TDO was not fully suppressed in ST subjects compared to ART-naïve, possibly because unlike IDO-1 and IDO-2, TDO seems to be a constitutively expressed gene; therefore, it is not induced or regulated by immune system signals [46]. Interestingly, levels of TDO were dramatically lower in EC compared to other study groups. Since TDO is mostly a hepatic enzyme, it may not account for Trp depletion observed in EC as this may potentially be mediated by other members of indolamine dioxygenase superfamily such as PrnB (the second enzyme in the pyrrolnitrin biosynthesis pathway) [47]. Our findings suggest a link between the distinctive Trp catabolism in EC and a consistently lower FOXO3a and TDO expression in these patients. Indeed, in EC both expression of TDO and FOXO3a which are glucocorticoid receptor targets are low when compared with any other HIV group or HS.   Polymorphisms on the promoter of the TDO gene that could affect expression and/or activity of TDO through glucocorticoid induction may explain the ability of EC to control oxidative stress and immuno-metabolism changes induced by HIV [48,49]. Of note, our results on TDO mRNA expression were obtained from PBMCs and this may be a limitation to the study.

An association between enhanced IDO activity in DCs and Treg induction has been previously reported in chronic hepatitis C infection [50]. Accordingly, recent studies showed that *in vitro* HIV-1 infection impairs the capacity of DCs to induce Tregs [51]. As IDO is intracellular and not secreted, the metabolic effects of IDO begin as inherently local signals stemming from CTLA-4 ligation with CD80/CD86 expressed by DCs [3,7,52,53]. Acute HIV-1 infection is associated with a large increase in TLR signaling and IFN concentrations through plasmacytoid DCs resulting in IDO upregulation [39]. In addition, IL-1β, IL-6, and TNF-α, were shown to induce IDO activity and Kyn production *in vitro* in the presence of LPS and IFNγ [2,54]. The former findings are not in line with our *ex vivo* observations. Indeed, we found elevated levels of IL-6, sCD40L, IFNγ and TNF-α in ART-naïve subjects compared to EC. However only sCD40L and TNF-α were positively correlated with IDO enzymatic activity and IDO-1 mRNA expression, suggesting DC-induced Trp catabolism in association with these elevated inflammatory factors may be important drivers of HIV-1 disease progression. In previous studies, neopterin, an independent prognostic factor for HIV disease outcome and immune activation was shown to correlate Trp degradation [10,11]. Alternatively, in our study we evaluated the correlation between Trp catabolism and IL-6 plasma levels which is the best marker of HIV-1 associated disease progression and mortality [55,56]. We observed a similar positive correlation of IDO activity (Kyn/Trp ratio) and IL-6 plasma levels suggesting that Trp catabolites may be considered as inflammation-related markers for HIV-1 disease progression. Indeed, it has been shown that the Kyn/Trp ratio independently predicts mortality in association with high gut epithelial cell apoptosis in HIV-1 infection[55].

Collectively, our results favor a model where IDO-induced Trp catabolism in association with elevated levels of TNF-α and sCD40L has a harmful effect on Th17/Treg balance fueling microbial translocation in HIV-1-infected patients. In contrast, while EC displayed (1) low plasma Trp levels similar to ART-naïve patients, they did so without elevated immunosuppressive Kyn levels and Th17/Treg balance disruption and (2) EC present with a dramatically low TDO expression when compared to other study groups. Given the immuno-metabolic role of IDO in maintaining chronic inflammation, our results provide evidence that novel immunotherapeutic strategies using IDO inhibitors will highly likely help reduce immune activation while enhancing immune control, that could in turn contribute to HIV-1 eradication.

## Supporting Information

Table S1 **Correlations between IDO enzymatic activity (Kyn/Trp ratio) and levels of inflammatory soluble factors implicated in IDO induction.** Associations between IDO activity (Kyn/Trp ratio) and plasmatic levels of IL-1β, IFNγ, sCD40L, TNF-α and IL-6 in ST: ART-successfully treated, ART-naïve, EC: elite controllers, and HS: healthy subjects. n=14 per study group. Pearson rank correlation was used for statistical analysis.
(DOCX)

Table S2 **Correlations between IDO-1 mRNA expression and levels of inflammatory soluble factors implicated in IDO induction.** Associations between IDO mRNA expression and plasmatic levels of IL-1β, IFNγ, sCD40L, TNF-α and IL-6 in ST: ART-successfully treated, ART-naïve, EC: elite controllers, and HS: healthy subjects. n=14 per study group. Pearson rank correlation was used for statistical analysis.
(DOCX)
