# Correction: Distinct Tryptophan Catabolism and Th17/Treg Balance in HIV Progressors and Elite Controllers

**DOI:** 10.1371/annotation/11698dd2-0bc1-4fe0-ad92-b161b5594e81

**Published:** 2014-01-09

**Authors:** Mohammad-Ali Jenabian, Mital Patel, Ido Kema, Cynthia Kanagaratham, Danuta Radzioch, Paméla Thébault, Réjean Lapointe, Cécile Tremblay, Norbert Gilmore, Petronela Ancuta, Jean-Pierre Routy

Errors occurred in Table 2. The correct values for the last row IL-6 (pg/ml) in Table 2 are incorrect. The correct values are: ST : 1.94±0.81, ART-Naïve: 3.61±0.71, EC: 1.37±0.30, HS: 1.26±0.27, p value: 0.0089

