# Supplementary material for: Distinct Tryptophan Catabolism and Th17/Treg Balance in HIV Progressors and Elite Controllers
Source: PLoS One. 2013 Oct 16;8(10):e78146. doi: 10.1371/journal.pone.0078146 (PMC3797729; doi:10.1371/journal.pone.0078146)
Supplement: Table S1 — Correlations between IDO enzymatic activity (Kyn/Trp ratio) and levels of inflammatory soluble factors implicated in IDO induction. Associations between IDO activity (Kyn/Trp ratio) and plasmatic levels of IL-1β, IFNγ, sCD40L, TNF-α and IL-6 in ST: ART-successfully treated, ART-naïve, EC: elite controllers, and HS: healthy subjects. n=14 per study group. Pearson rank correlation was used for statistical analysis. (DOCX) [file pone.0078146.s001.docx]

|  | **Kyn/Trp ratio** | | | | |
| --- | --- | --- | --- | --- | --- |
|  | **All**  **(n=56)** | **ST**  **(n=14)** | **ART-naïve**  **(n=14)** | **EC**  **(n=14)** | **HS**  **(n=14)** |
| **IL-1β** | *p* = 0.2920 | *p* = 0.5175 | *p* = 0.6616 | *p* = 0.9581 | *p* = 0.3663 |
|  | R = 0.1433 | R = -0.1890 | R = -0.2185 | R = 0.0155 | R = -0.2616 |
| **IFN-γ** | *p* = 0.4320 | *p* = 0.0932 | *p* = 0.7889 | *p* = 0.9271 | *p* = 0.4804 |
|  | R = -0.1071 | R = 0.4659 | R = -0.0788 | R = 0.0103 | R = -0.2057 |
| **sCD40L** | *p* = 0.0028 | *p* = 0.5645 | *p* = 0.0270 | *p* = 0.3685 | *p* = 0.0435 |
|  | R = 0.3916 | R = 0.1686 | R = 0.5880 | R = -0.2604 | R = -0.5457 |
| **TNF-α** | p < 0.0001 | p = 0.8787 | p = 0.9259 | p = 0.1090 | p = 0.3174 |
|  | R = 0.5312 | R = -0.0471 | R = -0.0274 | R = 0.4471 | R = -0.2884 |
| **IL-6** | p = 0.0020 | p = 0.3145 | p = 0.3662 | p = 0.0090 | p = 0.3748 |
|  | R = 0.5312 | R = -0.2900 | R = 0.2616 | R = 0.6684 | R = -0.2571 |
