# Supplementary material for: Distinct Tryptophan Catabolism and Th17/Treg Balance in HIV Progressors and Elite Controllers
Source: PLoS One. 2013 Oct 16;8(10):e78146. doi: 10.1371/journal.pone.0078146 (PMC3797729; doi:10.1371/journal.pone.0078146)
Supplement: Table S2 — Correlations between IDO-1 mRNA expression and levels of inflammatory soluble factors implicated in IDO induction. Associations between IDO mRNA expression and plasmatic levels of IL-1β, IFNγ, sCD40L, TNF-α and IL-6 in ST: ART-successfully treated, ART-naïve, EC: elite controllers, and HS: healthy subjects. n=14 per study group. Pearson rank correlation was used for statistical analysis. (DOCX) [file pone.0078146.s002.docx]

|  | **IDO-1 mRNA** | | | | |
| --- | --- | --- | --- | --- | --- |
|  | **All**  **(n=56)** | **ST**  **(n=14)** | **ART-naïve**  **(n=14)** | **EC**  **(n=14)** | **HS**  **(n=14)** |
| **IL-1β** | *p* = 0.9397 | *p* = 0.2808 | *p* = 0.3741 | *p* = 0.7398 | *p* = 0.9397 |
|  | R = -0.0108 | R = -0.3572 | R = 0.2575 | R = -0.0977 | R = -0.0108 |
| **IFN-γ** | *p* = 0.8693 | *p* = 0.6802 | *p* = 0.5806 | *p* = 0.9183 | *p* = 0.5842 |
|  | R = -0.0234 | R = 0.1405 | R = -0.1618 | R = 0.0302 | R = -0.1676 |
| **sCD40L** | *p* = 0.0015 | *p* = 0.5439 | *p* = 0.0175 | *p* = 0.7378 | *p* = 0.5883 |
|  | R = 0.4289 | R = -0.2057 | R = 0.6223 | R = -0.0984 | R = 0.1658 |
| **TNF-α** | *p* = 0.0325 | *p* = 0.4788 | *p* = 0.3453 | *p* = 0.8502 | *p* = 0.6279 |
|  | R = 0.2969 | R = 0.2391 | R = -0.2728 | R = -0.0556 | R = 0.1487 |
| **IL-6** | *p* < 0.0001 | *p* = 0.2556 | *p* = 0.0181 | *p* = 0.4259 | *p* = 0.1330 |
|  | R = 0.5939 | R = -0.3751 | R = 0.6196 | R = -0.2315 | R = 0.4394 |
